# A qualitative exploration of the barriers and facilitators to early lower limb assessment and onward referral for specialist treatment for patients with venous ulceration

**DOI:** 10.1111/iwj.70071

**Published:** 2025-01-12

**Authors:** Layla Bolton Saghdaoui, Smaragda Lampridou, Alun Huw Davies, Sarah Onida, Mary Wells

**Affiliations:** ^1^ Section of Vascular Surgery—Department of Surgery and Cancer Imperial College London/Imperial College Healthcare NHS Trust Charing Cross Hospital London UK; ^2^ Directorate of Nursing, Imperial College Healthcare NHS Trust/Imperial College London Education Centre Charing Cross Hospital London UK

**Keywords:** assessment, care pathways, clinical pathways, hospital referral, venous leg ulceration

## Abstract

Guidance for venous leg ulceration (VLU) recommends compression therapy and early referral for specialist vascular assessment within two weeks. Few patients receive timely assessment and referral. Reasons for this are unclear. The aim of this work was to explore nurses' perceptions of the barriers and facilitators to early assessment of VLU and referral for specialist treatment. One‐to‐one semi‐structured interviews explored experiences caring for and referring patients with VLU to a vascular specialist. Maximum variation sampling and inductive thematic analysis were used. Eighteen nurses participated. Findings suggest junior nurses lack knowledge and confidence to care for VLU and often revert to a ‘task‐based’ approach, exacerbated by staff shortages and limited training. Because VLU occurs in the context of competing conditions and pressures, comprehensive assessments are missed, and the need for referral is not established or prioritised. Supporting patients to self‐manage is seen as a possible solution. Nurses reported disjointed pathways between primary and secondary care, compounded by poor MDT collaboration, ineffective communication systems and inadequate data sharing. Consequently, when the need for referral is established, communicating this between healthcare organisations is complex. Organisational and behavioural barriers impact nurses' ability to promote timely referral. Further exploration with patients and other healthcare professionals is needed.

## 1 INTRODUCTION

Venous leg ulceration (VLU) is a major health problem. Whilst such wounds commonly develop due to superficial or deep vein incompetence (sometimes secondary to deep vein thrombosis), VLUs are also caused by functional venous disease. When left untreated, this leads to inefficient venous drainage from the lower limbs, causing venous hypertension and, in turn, the development of skin damage, later resulting in ulceration.^1^ Whilst between 60% and 80% of all lower limb wounds are venous in nature, prevalence and incidence are difficult to estimate due to the significant heterogeneity across the current body of literature.^2^

Patients with active VLU experience chronic pain, reduced mobility and isolation. Many see a decline in their mental health and ability to perform activities of daily living, and unfortunately, once VLU is healed, the burden does not disappear, as approximately 70% of patients experience recurrence within 12 months.^2^, ^3^ In addition to the burden on quality of life, VLU costs the NHS more than £2 billion per year and utilises approximately 2% of the annual healthcare budget in Western countries.^4^ A significant cost relates to the workload burden of community nurses, who spend up to 50% of their time managing this patient population.^5^ For instance, in Hull and East Yorkshire, 151 000 nursing hours per annum were required to facilitate dressings for VLU, the full‐time equivalent of 88.5 nurses.^6^

Evidence‐based treatment for VLU includes the diagnosis of venous disease, exclusion of peripheral arterial disease, compression therapy and endovenous ablation.^7^, ^8^ Most venous leg ulcers are managed by nurses in primary care, where peripheral arterial disease can be excluded using clinical assessment and Ankle‐Brachial Pressure Index (ABPI) measurements. Compression therapy can also be delivered effectively in this setting.^5^, ^9^ However, at present, gold‐standard diagnostic testing to identify venous disease (duplex ultrasound) and evidence‐based therapy (endovenous ablation) are predominantly delivered by vascular surgical teams in hospitals.^7^, ^8^

To access such services, the National Institute for Health and Care Excellence (NICE) published guidance in 2013 recommending that patients with suspected VLU persisting for more than 2 weeks be referred for specialist assessment by a vascular service.^10^ Following this, in 2018, findings from the Early Venous Reflux Ablation (EVRA) trial^11^ demonstrated that early endovenous treatment is a cost‐effective strategy that increases healing rates, increases ulcer‐free time and reduces recurrence.^12^ This provided additional weight to the NICE referral guidance, and to ensure patients have access to endovenous intervention in a timely manner, the Venous Forum and The National Wound Care Strategy Program (NWCSP) published guidance supporting early referral for comprehensive vascular assessment taking place within 14 days of presentation.^13^, ^14^

Despite this evidence‐based guidance, only a small proportion of eligible patients receive appropriate assessment and referral. This was demonstrated in early 2018 through an evaluation of a UK‐based data set of patients with venous ulceration. This study concluded there had been no meaningful change in venous leg ulcer management in primary care in line with guideline recommendations.^10^

Although reasons for this are unclear, following this, the ‘Burden of Wounds’ Study, published in 2020, found significant shortcomings in the timely assessment and diagnosis of chronic wounds, with 78% of patients with suspected VLU not receiving a Doppler as part of the early assessment.^15^ Moreover, a UK survey of general practitioners (GPs) and community nurses revealed poor adherence to NICE guidance, with only 37% of VLU patients being referred to a vascular centre in less than 6 weeks.^16^ A subsequent survey found the median referral time in the UK was approximately 8 weeks, significantly longer than the NICE 2‐week recommendation.^17^ Considering the poor assessment and referral rates, it is no surprise that there are disparities in the number of patients receiving treatment for venous insufficiency throughout Europe, with the UK having the lowest count of endovenous procedures.^18^ These shortcomings in venous leg ulcer care are a persistent issue across the NHS. Although the care pathway for patients with VLU continued as normal throughout the COVID‐19 pandemic, only 15% received a Doppler as part of early assessment, and 35% fewer patients were referred to a hospital specialist.^19^ Unfortunately, it is widely accepted that since then, there has not been a sustained improvement in care, and considerable variations in clinical practice still lead to delays in diagnosis and inappropriate treatment plans.^20^

As shown in the current literature, poor adherence to healthcare guidance is common and influenced by organisational challenges and clinician/patient behaviour. Despite best intentions, staff often describe lacking the skills and tools to deliver recommendations.^21^ In the case of VLU pathways, particular organisational barriers may hinder the process of referral. For example, whilst community nurses are the main caregivers of patients with VLU, the survey of UK‐based staff reported that 73% of nurses could not refer directly to secondary care.^17^

Evidence suggests that interventions requiring behaviour change in healthcare staff often experience low uptake and face implementation challenges.^22^ This can be due to poor planning and lack of relevant intervention framework or behavioural theory. Consequently, there is often limited evaluation of how an intervention translates into real‐world clinical practice.^23^

To tackle this issue, enhancing referral rates has been identified as a key research and clinical priority in the UK by the 2021 James Lind Alliance Vascular Priority Setting Partnership and the 2023/2024 Commissioning for Quality and Innovation (CQUIN) scheme.^24^, ^25^ Addressing the clinical priority and aiding implementation of the CQUIN, the NWCSP commissioned the nationwide programme, ‘Transforming Wound Care’. This program aims to optimise healing and the impact of chronic wounds by developing clinical recommendations. However, despite the national agenda in this area, there is still limited evidence regarding how patients access care for ulceration and the cause of delays. The Medical Research Council's guidance on developing and implementing healthcare interventions advises that prior to implementation, there should be a sound understanding of key uncertainties and the context in which a potential intervention will lie. This information should be sought from the literature and qualitative exploration with key stakeholders.^26^ To do this, this study aimed to explore the barriers and facilitators to early assessment and referral through understanding the views and experiences of nurses, who have a major role in assessing and caring for patients with venous ulcers across a range of settings.

## 2 MATERIALS AND METHODS

### 2.1 Study design

#### 2.1.1 Methodological orientation

A qualitative study involving nurses working in primary and secondary care was conducted over a 1‐year period from October 2020. Qualitative methodology is useful when there is a need to explore real‐world situations in a detailed manner.^27^ As such, it was well placed to aid our understanding of the referral process beyond the survey and retrospective data that was already available.^16^ To aid the study design, input was sought from a clinical advisory group of three registered nurses from distinct clinical settings who had different levels of experience working with patients living with VLU. Their input during the project's preparation phase influenced the choice to conduct individual semi‐structured interviews rather than focus groups, as they felt that larger group settings would hinder their ability to express their thoughts freely.

Ethical approval was granted by the London—Surrey Borders Research Ethics Committee (REC reference: 20/LO/0247/IRAS ID: 270915). Written consent was gained before each interview.

#### 2.1.2 Participant selection and setting

Participants were recruited from primary and secondary care settings and through professional social media channels. Settings included a major London acute teaching hospital trust, a local community trust, two Royal College of Nursing specialist interest groups, a UK tissue viability network and a UK lower limb clinicians' network. Of those willing to participate, a screening proforma was completed to enable a strategy of maximum variation sampling to ensure a diverse group of registered nurses and allow for comparative analysis.^28^ Purposive sampling characteristics included ethnic background, level of clinical seniority and clinical practice area (e.g., GP practice, district nurse practice, primary care wound clinic, tissue viability clinic, vascular surgery). Specific inclusion criteria required nurses to have experience caring for patients with VLU within the last 6 months.

As this study was conducted during the COVID‐19 Pandemic, adhering to regulations at the time, nurses were invited to complete an individual face‐to‐face, over the telephone or virtual interview via Microsoft Teams or Zoom.

#### 2.1.3 Data collection

Individual semi‐structured interviews were chosen for this study following feedback from the clinical advisory group, which advised nurses may not feel comfortable speaking freely in a focus group. Where possible, interviews took place outside of the participants' current working environment.

Only the researcher (LBS) and the participant were present at each interview, and no repeat interviews took place. The interview topic guide (Appendix A) was informed and piloted by the advisory group and patient and public involvement activities and focused on the following key topics:

1. The assessment and management of VLU
2. Referring patients
3. Barriers and facilitators to guideline recommend treatment for VLU

Taking place between October and February 2020, 18 interviews were completed, lasting an average of 47 min. Recruitment and data collection continued until the research team considered that sufficient data (defined as the depth, diversity and adequacy of the data) were obtained to answer the research questions.^29^ To aid data analysis, field notes were collected, and interviews were recorded digitally and transcribed verbatim. Transcripts were compared with the recordings to check accuracy, and notes were made to identify initial impressions. Transcripts were then uploaded to the data management software QSR N‐VIVO Version 14.

#### 2.1.4 Data analysis

Interviews were analysed inductively, using a thematic approach, as set out by Braun and Clarke.^30^ Each interview went through two rounds of coding. To start, this included familiarisation and noting down initial thoughts. The second round involved initial code generation,^31^ whereby a code was assigned to each line or section of each transcript, representing an important subject or meaning within a sentence or paragraph.^31^ Throughout the analysis process, visual diagrams were used to map codes into themes and combine those with similar meanings. Visual diagrams were also used to separate codes into barriers and facilitators to care. Participant recruitment and analysis were carried out simultaneously to allow for constant comparison and the identification of similarities, differences or patterns within the data. This also allowed authors to ascertain when data saturation had been achieved.^31^ When coding and categorising incoming data into themes, new themes were compared with those already developed.^29^ Once core themes were established, all transcripts were re‐reviewed to explore variations between the views of nurses in primary care versus secondary care and the views of senior and junior nurses. Once themes had been developed, they were presented to the study advisory group and patient and public involvement representatives to ensure the theme labels were representative of the study data.

### 2.2 Researcher team and reflexivity

The first author of this manuscript (LBS, female, vascular nurse) was the primary researcher, conducting interviews and performing data analysis. LBS did not directly work with any study participants, and each was informed of her research and clinical background prior to taking part in an interview.

LBS's experience working with VLU patients presented a potential for bias when conducting interviews and analysis. For example, LBS may have had a preconceived perception of the barriers to referral and the current pathway and, as such, may have focussed on areas she believed to be relevant or ask leading questions. To address this, the first two transcripts were reviewed to identify any leading questions and if there was a particular focus on certain topics. This was then discussed with a second author (SL) who does not work with VLU patients. In this meeting, potentially missed opportunities to explore certain topics were highlighted and incorporated into subsequent interviews. Additionally, a reflective journal was kept whilst conducting interviews and analysis. In these journals, LBS documented her thought process when reading transcripts, themes of importance and subjects needing further exploration and reflected on her interview technique. These journals were reviewed with the study team (SL and MW) to identify potential bias in interviews and the analysis process. Potential codes and themes were also reviewed and revised with authors SL and MW, neither of whom had any contact with participants.

## 3 RESULTS

### 3.1 Participant characteristics

Twenty‐two nurses expressed an interest in the study. Four nurses did not participate due to time pressures. Nurses working in both primary (8) and secondary care (10) settings participated, and all but one (a participant from an Asian background) described themselves as White British. Nurses represented clinical services from across the UK, including Northwest England, Yorkshire, The Midlands, East of England, the Channel Islands, Scotland, Southeast and Southwest England and London. Participant characteristics can be seen in Table 1.

**TABLE 1:** Participant characteristics.

Professional titles	
Tissue viability nurse *(primary care)*	5
Tissue viability nurse (*secondary care setting*)	1
Vascular clinical nurse specialist/advanced nurse practitioner (*secondary care setting*)	6
District/community nurse	3
Leg ulcer nurse (*primary care setting*)	2
General/family practice nurse	1
UK AfC professional banding scale^a^	
Band 5	3
Band 6	5
Band 7	7
Band 8	3
Nationality	
White British	19
Asian	1
Years of experience working with VLU	
0–5 years	3
5–10 years	5
10–20 years	5
20+ years	5

Abbreviation: VLU, venous leg ulceration.

^a^ The system Agenda for Change (AfC) categorises nursing roles in the UK and is comprised of nine bands (1–9). Each band is associated with specific abilities and responsibilities, and an entry‐level registered nursing post starts at band 5.^36^

### 3.2 Main findings

**FIGURE 1:**
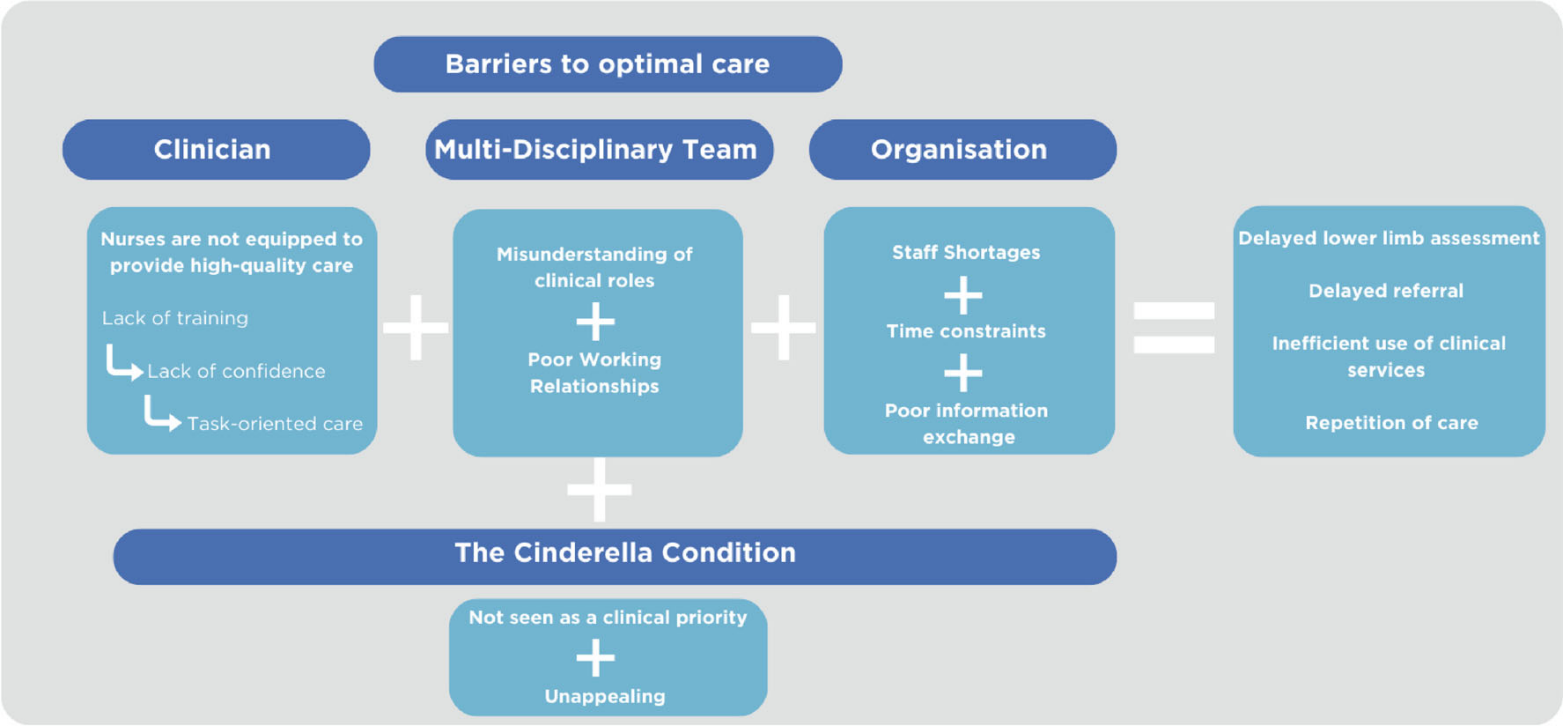
Barriers to optimal care.

The data across all transcripts clearly showed that current processes for accessing early assessment and referral are lengthy and complex. Barriers and facilitators to referral could be explained in four key themes: being equipped, multi‐disciplinary team (MDT) working, organisational limitations and ‘the Cinderella condition’. Figure 1 depicts these four key themes, and although each one includes unique barriers and facilitators, several are interlinked. Barriers for clinicians include a lack of training and confidence, which leaves them feeling unequipped to provide VLU care and results in a tendency for task‐oriented management. The second theme, *MDT working*, illustrates how poorly defined clinical roles and ineffective working relationships hinder a shared care approach. *Organisational Limitations* describes how systems and process barriers, such as staff shortages and time constraints, make implementing early assessment and referral challenging. Together, the combination of clinician, MDT, and organisational factors all influence nurses' ability to provide guideline‐recommended VLU care. Compounding these barriers is the perception that VLU is not seen as a priority in the context of competing clinical pressures, resulting in the final theme, ‘The Cinderella Condition’.

### 3.3 Being equipped

Both junior and senior nurses emphasised that being well‐equipped to provide high‐quality care for patients with VLU included having sufficient training and skills. Unfortunately, across the current workforce, the lack of training and knowledge was particularly prominent amongst junior nursing staff and included gaps in knowledge related to initial assessments, compression bandaging, complex wound care and the common underlying causes of ulceration.

N14: it is a difficult one because I don't think we, we're given enough, um, training … we learn how to doppler and use the compression therapy appropriately. Um, and that's the only education you get… then that information's never updated so people are just going by what they've learnt.

[District Nurse]

N11: I can't remember ever being taught anything about leg ulcers when I was at uni and studying my nursing. Uh, um, and even when we did all ANP [Advanced Nursing Practice] and different conditions, there was never anything about leg ulceration.

[Vascular Clinical Nurse Specialist]

These gaps in knowledge often meant that nurses felt unable to complete an initial assessment or identify a possible underlying cause for ulceration. Consequently, they were then unable to progress to the next stage, where onward referral would be considered. Some participants were particularly uncomfortable with initial assessment and clinical decision‐making as they felt it was aligned with making a diagnosis, which some felt was not a nurse's role.

N01: ‘it's quite a decision‐making process to, you know… You're making a differential diagnosis, which nurses don't tend to do, um, in, in any other field, really’.

[Lead Tissue Viability Nurse—Primary care]

N12: ‘Referral is a bit of, in some ways, a bit of a stumbling block. I think, in a way, we're, sort of, expecting practitioners that aren't used to referring people to refer people. Which causes anxiety and uncertainty’.

[Vascular Clinical Nurse Specialist]

Nurses often used strong and emotive words to describe concerns about both decision‐making and compression bandaging, including ‘nervousness’, ‘fear’, being ‘scared’ or ‘needing to be brave’. Nurses perceived these feelings stemmed from inadequate training and experience, resulting in a diminished sense of confidence. Consequently, senior nurses reported that juniors often fell into a pattern of ‘task‐based care’.

N01: ‘it's [referral] missed through the general task‐orientated care, and they're not thinking, okay, this isn't healing… Its [venous ulceration] scares the living daylights out of them … it's about being brave, and you need experience to be brave to make that decision’.

[Lead Tissue Viability Nurse—Primary care]

N07: ‘They don't have that background knowledge, and they probably don't have the confidence’.

[Community Matron for Tissue Viability]

This caused delays in referral as junior staff had to wait for reassurance or permission from senior clinicians.

N14: ‘people are just going every week, every, every, twice a week to people taking the bandaging off, washing their legs, dressing it, putting the bandaging back on, and not stopping to think actually, this is a waste of… It's not a waste of time but it could actually be treated better’.

[District Nurse]

N12: ‘it's knowing that it's all right to refer people. That, you know, nothing dreadful's going to come of it’.

[Vascular Clinical Nurse Specialist]

### 3.4 MDT working

In addition to being unequipped to care for patients with VLU, participants also reported issues defining clinical roles and working effectively as an MDT. Breaking this down, two sub‐themes were evident. The first is ‘Whose job is it?’ where participants expressed varied views regarding the types of clinical responsibilities that should be allocated to specific professional roles amongst the MDT. This was particularly evident when considering what clinical speciality should assume overall responsibility for this patient population. The second sub‐theme is ‘Working relationships’, which explores how working as a team inevitably affects the patient pathway and patient experience.

#### 3.4.1 ‘Whose job is it?’

When considering day‐to‐day wound care and compression therapy, all participants suggested that the care of patients with VLU should be predominantly led by nurses. Nurse‐led care was seen positively, as respondents perceived nurses as capable of offering holistic assessments, health promotion and emotional support.

N07: ‘I think because it's seen as a wound, um, that's what nurses do’.

[Community Matron for Tissue Viability]

However, their views differed in relation to what type of nurse should be responsible for wound care and compression therapy on a day‐to‐day basis. Some reported a lack of clarity and potential duplication between specialist nursing roles, such as tissue viability nurses, vascular nurse specialists and district nurses.

N02: ‘the tissue viability nurses should be able to do them, but I also think I'm not sure from a vascular ward point of view, whether they deem us in theory able to do it’.

[Vascular Clinical Nurse Specialist]

N14: ‘If the person needs it, we'll do it, but it's not fair to expect the district nurses just to pick up everything… in district nursing we feel like we were getting dumped on. Do you know? Oh, we don't really know what to do with this, we'll just, we'll just send it to the district nurses’.

[District Nurse]

Whilst nurses in all settings considered it appropriate for wound management to be part of the nursing role, the ability to formulate a high‐quality treatment plan, including the application of compression therapy, was perceived as a specialist skill, outside the scope of a general band five nurse working in primary care. For example, interpreting the results of a duplex ultrasound and establishing the need for venous intervention or sharp wound debridement was seen to be a specialist skill. Whilst such skills were thought to be within the scope of nurses, this was only appropriate after further training.

N16: ‘it should be someone that was trained in doing vascular. I don't know. That's like a specialist role isn't it’.

[Primary care—Practice Nurse]

N11: ‘I don't think it needs to be consultant‐led. I think it can be led by somebody who is an experienced vascular nurse or leg ulcer nurse, um, who's trained specifically in venous leg ulceration’.

[Vascular Clinical Nurse Specialist]

#### 3.4.2 Working relationships

Strong relationships between members of the MDT, particularly across primary and secondary care, were seen to be a facilitator of referral, with some nurses believing that good teamwork provided a ‘safety net’ for patients. Unfortunately, it was uncommon for participants to report having strong working relationships, connected services or multi‐professional working between primary care and vascular services.

N03: I think it [closer MDT working] would be a positive impact, ‘cause I think the patients would get a referral more quickly and have a quicker, um, intervention, to be honest’.

[Vascular Clinical Nurse Specialist]

N04: ‘part of the problem, just purely because we're [healthcare professionals] not talking… We are not sharing and talking to other professionals. The patient's in the middle’.

[Tissue Viability Nurse—Secondary care]

Similarly, the working relationship between district nurses and GPs often appeared to be strained, and many community nurses reported feelings of ‘frustration’ and ‘mistrust’. Whilst district nurses felt responsible for patient care on a day‐to‐day basis, they often reported being unable to action a referral to specialist services without GP involvement. This was reported as a barrier to providing timely and effective VLU care.

N17: ‘it, it can be very frustrating, especially if you've got a GP that drags their feet and doesn't put the referral through, um, and you have to chase it and chase it’.

[Tissue Viability Nurse—Primary care]

N05: ‘quite a lot of animosity between, it's like an us and them relationship at the moment and sometimes it's not nice and GPs and practice nurses get frustrated with us, we get frustrated with them’

[District Nurse]

In contrast, primary care nurses felt the nurse‐to‐nurse relationships between practice, district and TVNs were strong. Although established connections between vascular nurse specialists working in secondary care and community nurses were uncommonly reported, they facilitated good practice in departments where they existed. For junior nurses, these relationships helped to facilitate clinical assessments, care planning and training, and nurses reported feeling confident referring to one another.

N05: ‘TVNs are really, really useful. They're very knowledgeable. So, they do give us, you know, a good plan following them seeing patient and doing their assessment as well. So, yes, I really do like appreciate our tissue viability service’.

[District Nurse]

### 3.5 Organisational limitations

Adding an additional level of complexity to the care of VLU, the organisational limitations reported by nurses could be explained by three subthemes: ‘systems and process barriers’, ‘information exchange’ and ‘self‐management’. Nurses highlighted various constraints, including challenges with staffing, high clinical workloads and inadequate clinical workspaces. Additionally, obstacles such as limited appointment times and the requirement for GP approval prior to making a referral were seen as direct barriers affecting care. Poor communication was reported as a significant issue. However, participants felt that advancements in technology were improving information transfer between primary and secondary care. Working to address such limitations, nurses reported a move towards empowering patients to self‐manage their wounds.

#### 3.5.1 System and process barriers

Staff shortages and high staff turnover were raised as an issue by all interviewees, and this particularly affected nurses in terms of their ability to carry out an initial assessment. Nurses reported that new staff rarely had the necessary skills to deliver VLU care. Unfortunately, due to their clinical workload, senior nurses felt they were limited in their availability to provide the necessary training.

N15: ‘Well, for now, our service is quite depleted’.

[Leg Ulcer Clinic Nurse — Primary Care]

N07: ‘there's vacancies out there with district nursing. So, it's a time issue … staff have left that are competent and then because we haven't done the theoretical training then the people coming in behind then aren't competent in doing it’.

[District Nurse]

When exploring barriers to early assessment in primary care, nurses explained that appointment slots in GP surgeries or community clinics were not long enough to carry out a full holistic assessment. Additionally, in both primary and secondary care, limited facilities and estates also posed challenges.

Many nurses remarked on the lack of appropriate clinical space and equipment to undertake optimal assessment and deliver wound care.

N09: ‘Appointments are only ten minutes, so that's a failure of the system, if they want to see a leg ulcer patient, they've got to book them for a couple of appointments’.

[Advanced Vascular Nurse Practitioner]

N02: ‘Over the years, we've very much deskilled and taken the resources away. So, we, so we don't have the baths anymore. We don't have the nurses with the skills anymore’.

[Vascular Clinical Nurse Specialist]

The most frequently mentioned systems barrier to referral was primary care nurses' inability to refer directly to specialist services such as vascular surgery. This was often due to regulations set by local clinical commissioning groups requiring referrals to come from a GP to ensure payment for services. This affected both junior and senior nurses, including tissue viability nurses and lead district nurses who had to refer via the patient's GP practice.

N05: ‘I just wish it [referral] could be a lot easier and that it could be done at our level of district nurses and community staff nurses’.

[District Nurse]

#### 3.5.2 Information exchange

Challenges with communication appeared to be common across all participants, and nurses felt that poor communication considerably affected VU care and onward referral. Communication between clinicians in primary and secondary care was reported to be particularly difficult, and nurses felt this disconnect caused delays, repetitiveness, and inefficiencies. When describing the current pathway, primary care nurses explained that when a patient is seen in a specialist service, communication of the outcomes is often shared with the GP and rarely fed back to community nurses. As a result, they reported being unable to promptly action advice or care plans.

Secondary care nurses reported difficulties getting in touch with primary care teams to clarify what clinical care plans were already in place and explained that this could mean they recommended dressings or wound care that had already been tried and shown to be ineffective.

N06: ‘if you've [the nurse] done the referral, they don't refer back to us. It always goes back to the GP’.

[District Nurse]

N12: ‘you see them and make a recommendation, it may well be that that's already been tried…. So, all you're doing is, kind of, repeating’.

[Vascular Clinical Nurse Specialist]

When exploring how nurses felt communication could be improved, many suggested that electronic systems should be joined up and new and emerging technology should be utilised.

N05: ‘Being able to directly refer through, um, electronic systems. I, I would really love for everybody to be on the same system’.

[District Nurse]

#### 3.5.3 ‘Self‐management’

Several participants raised the potential for patients to take on aspects of their own care and felt that this could help to address organisational challenges such as staff shortages. Nurses often used the term ‘self‐management’ or ‘supportive self‐management’ when discussing the move towards empowering patients to participate or take ownership of wound care. Many felt it gave them more time to focus on alternative tasks such as ongoing assessment and that this had become more acceptable due to the accessibility of alternative compression hosiery options such as wrap‐banding and hosiery kits.

N01: ‘we need to work towards a self‐care model … cause then you release time, don't you’.

[Lead Tissue Viability Nurse — Primary care]

N18: ‘I think they, then, feel empowered and then they want to do it, or their relatives want to do it.’

[Leg Ulcer Clinic Nurse — Primary Care]

When asked what they meant by self‐management, nurses suggested that this was about the patient's ability to clean their wound and apply dressings or compression independently. However, not all believed patients were best placed to carry out wound management, and there was a belief from most that self‐management was only for ‘the right patient’. The right patient was perceived to be someone willing to engage in their care and able to identify ‘red flags’ or concerns.

N06: ‘The wraps, they can do themselves. They can put them on, take them off’.

[District Nurse]

N16: ‘It very much depends on the patient, really. And I think sometimes we have a number of patients who are willing to engage, um, and want to be self‐caring, other patients like to come to clinic.’

[Primary care — Practice Nurse]

### 3.6 ‘The Cinderella condition’

The organisational limitations identified often occur in a context in which VU is not perceived as a priority, and several participants labelled VU as ‘the Cinderella condition’. Clinicians specialising in VU expressed a perception that healthcare professionals working in generalist settings, such as district nurses and GPs, view venous ulcers as unpleasant. When discussing VU, participants often employed negative language like ‘smelly’ and made comments such as ‘we want to get them off the books’ and ‘it's a burden’. Consequently, senior nurses faced difficulties encouraging junior nurses to be ‘excited about leg ulcers’, and many commented that they think nurses avoid training in leg ulcer care.

N08: often leg ulcers are seen as the Cinderella. You know, they're smelly, they're dirty, they're wet.

[Advanced Vascular Nurse Practitioner]

N10: ‘if you do the training, you get stuck doing all the wounds’.

[Advanced Vascular Nurse Practitioner]

It was also common for nurses to report that patients with VU were not seen as a priority when compared to other health conditions. In primary care and district nursing services, this was seen to particularly affect initial assessment and onward referral, as higher‐risk patients with other conditions took priority.

N11: ‘they're dealt a poor hand currently, I think. I think, you know, the, they're patients that are almost bottom of the pile, um, because things aren't life threatening. Um, and it's seen as a leg ulcer and you can live with it and, and get on with it’.

[Vascular Clinical Nurse Specialist]

N01: ‘Um, it [assessment] can be delayed, and it's put off. If they've got an allocated visit or end‐of‐life care, it takes priority’.

[Tissue Viability Nurse — Primary care]

When senior nurses described the process of managing wound care services, they also reported a perception that VU patients were deemed as low in priority by clinical and administrative management teams in primary care. This view was felt to result in a lack of funding and commissioning for VU services.

N18: ‘I don't think they [primary care staff] have the funds to do any form of Doppler. And I don't know whether they've ever seen a lower limb assessment’.

[Leg Ulcer Clinic Nurse — Primary Care]

N11: ‘There's no extra funding that comes with it. Um, it's just, I think they [GPs] just see it as a burden’.

[Vascular Clinical Nurse Specialist]

## 4 DISCUSSION

This study aimed to identify nurses' perceptions of the barriers and facilitators to the assessment of suspected venous ulceration and onward referral to vascular surgery. The findings suggest that several barriers exist, which cause delays to referral and mean that current clinical guidelines cannot be met. Many of the perceived reasons for delays in referral stemmed from resource limitations, including a lack of training for staff, time pressures, space and equipment and poor staffing levels. Furthermore, nurses felt that disconnected pathways between MDT departments, particularly across primary and secondary care, impeded timely assessment and management. The recent Leg Ulcer Pathway Acceleration (LUPA) study reported similar findings from their qualitative analysis. Their evaluation of the development of a structured pathway for referral found that poor staffing levels, high staff turnover and fragmented pathways between primary and secondary care were common barriers to referral.^32^ Another commonality across the two studies was that information exchange between primary and secondary care was particularly challenging.^32^ Nurses in the current study reported that they were often unaware of the treatment plan set by other MDT members, leading to delays, repetitiveness and inefficiencies. These issues are also apparent within the theme, *MDT Working*, where nurses described not receiving timely information from GP colleagues as the primary cause of strained relationships. This was particularly problematic for community nurses who are unable to refer directly to secondary care and therefore relied on GPs to be ‘middleman’. A survey of primary care staff caring for patients with VU found similar practices, with 73% of respondents reporting that only GPs could make a referral.^16^ To improve communication and information exchange, nurses in this study felt it would be helpful if they could be the main point of contact. They also expressed the need to improve electronic systems between primary and secondary care, adopting emerging technology where possible.

Organisational barriers were compounded by the perceptions, behaviours and confidence of nurses.

Due to high staff turnover and a lack of available training for new and junior staff, community nurses often felt ill‐equipped to care for patients with VU. They also reported time constraints and the lack of appropriate clinical space. Without adequate preparation, nurses did not have the skills or confidence to perform the required lower limb assessment and establish the need for onward referral.

In the context of these system and process barriers, nurses seemed more likely to focus on wound dressings alone, describing a task‐oriented rather than a holistic approach to care. To address resource challenges, some participants discussed encouraging selected patients to self‐manage. By empowering people to take ownership of their wound care and, for example, manage their own velcro wrap bandages, nurses felt they could spend more time on ongoing assessments instead of dressing applications.

Other studies have also reported challenges to ensuring nurses are adequately trained in VLU competencies.^33^, ^34^ In the NWCSP evaluation, education was thought to be critical to effective wound care and patient management, but staff struggled to complete the training offered to them. Moreover, workload pressures meant that senior staff members were unable to commit the time to observe trainees.^13^ The NWCSP evaluation described a similar lack of engagement in education and training across different healthcare professional roles, with findings supporting our perception of VU being a ‘Cinderella condition’.^13^

Although nurses in this study focussed considerably more on barriers than on facilitators to early assessment and referral, many emphasised that strong working relationships across the MDT positively impacted the timely management of VUs. Similarly, well‐established nurse‐to‐nurse relationships between community nurses, TVN's and Vascular Nurse Specialist helped to facilitate assessment, care planning, referral and training.

Throughout this qualitative exploration, it is evident that many of the themes described in this work interconnect and, in turn, make the care pathway of this patient population complex. The combination of organisational and system barriers, perceptions and behaviours of nurses and other clinicians make it likely that interventions to improve referral need to be informed by theories of complex intervention development and behaviour change.

### 4.1 Limitations

This study provides a detailed insight into the care of patients with VU. However, it only represents the views of nurses. The care of VU involves several members of the MDT, and the experiences of all are needed to fully understand the current barriers and facilitators to early assessment and referral. Most importantly, to fully understand the impact of such barriers and facilitators on clinical outcomes and patient experience, the views of patients must be explored. Recruitment for this study was predominantly through self‐referral, which presents a potential bias as the nurses who volunteered to participate are more likely to have a special interest in VU.

This exploratory study aimed to inform more detailed qualitative work, and as a result, it was not designed or analysed using an underpinning theory. As seen in the results, the study found that certain organisational barriers, personal beliefs and behaviours of nurses, such as ‘task‐based care’, affect their practice. Using an appropriate behavioural theory, such as the theoretical domains framework could have helped to categorise organisational and behavioural barriers systematically and, in turn, identify ways to address them.^35^

## 5 CONCLUSION

Behaviours, working relationships, organisational barriers and clinician personal beliefs impact upon nurses' ability to provide optimal care. Facilitators for timely assessment and referral include strong working relationships between the multi‐disciplinary team, effective information sharing and supported self‐management. The barriers described in this study suggest that any intervention aimed at improving the timeliness of assessment and referral needs to address multiple behaviours, perceptions and system‐wide issues. To do so, such interventions could include integration of shared electronic health systems across primary and secondary care, development of MDT working relationships and a focus on clinician training. To do this effectively, the use of a complex intervention framework and underpinning behavioural theory is likely to be required. Although understanding the perspective of nurses is helpful, further exploration with patients and other healthcare professionals is also needed.

## FUNDING INFORMATION

Layla Bolton Saghdaoui and Smaragda Lampridou are funded by Health Education England (HEE)/National Institute for Health and Care Research (NIHR) for this research project. This work was also supported by Imperial Health Charity and NIHR Imperial BRC. The views expressed in this publication are those of the author(s) and not necessarily those of the NIHR, Imperial Health Charity or the UK Department of Health and Social Care.

## CONFLICT OF INTEREST STATEMENT

The authors declare no conflicts of interest.

## Data Availability

The ethical approvals for this work do not allow the raw data (interview transcripts) to be shared outside of the study team, as this could result in participants being identified.

## APPENDIX A TOPIC GUIDE/DRAFT QUESTIONS

### A.1 TOPIC GUIDE

The following topics will be used as an interview guide for nurses and have been developed based on the feedback gained from the study advisory group in the preparation phase for this project. Preparation included informal conversations with three registered nurses all currently working with venous leg ulcer patients. Nurses will be asked about their experiences of caring for venous leg ulcer patients, including the following key points:

**The assessment of wounds—** *How leg ulcers are assessed and diagnosed, delays and challenges experienced*.

When speaking to nurses with experience caring for leg ulceration, they expressed that the wound assessment and diagnosis can often be delayed. Under this topic heading, we aim to explore the reasons why.

**Referring patients/confidence in referring patients—** *Current systems for referral and experiences of referral will be explored*.

Nurses in the advisory group mentioned that they cannot currently directly refer patients to a vascular specialist, which causes delays. This topic will allow us to explore how they are currently managing this patient group and their views of alternative models of referral.

**Barriers and facilitators to treatment—** *What are nurses' experiences of factors which constrain or facilitate referral?*

Nurses in the advisory group explained that time constraints and poor communication can add to delays in referral. This topic will allow us to explore this in detail.

### A.2 DRAFT QUESTIONS

How often do you see patients with ulceration?

#### A.2.1. Referring patients/confidence in referring patients

Thinking about the MDT can you tell me what health professional you think a patient with venous ulceration should see?

What are some of the challenges you've faced when trying to refer a patient to a different department?

How confident do you feel filling out referral forms?

What are your thoughts on the type of information included in referrals you receive?

How useful do you think it is referring patients to vascular services?

#### A.2.2. The assessment of wounds

What is your experience of assessing wounds?

In your experience how are patients' ulcers diagnosed as being venous?

What are some of the challenges you face when trying to get the appropriate assessment for the diagnoses of an ulcer?

### A.3 BARRIERS AND FACILITATORS TO TREATMENT

Can you explain how patients are referred to your department?

Can you explain how your referral process works?

How do you feel about the time it takes for a referral to be processed?

How do you feel about the level of detail included in referrals you receive?

How do you feel about the time it takes to organise a referral?
